# Crystal structure of 2-methyl­amino-4-(6-methyl-4-oxo-4*H*-chromen-3-yl)-3-nitro­pyrano[3,2-*c*]chromen-5(4*H*)-one with an unknown solvate

**DOI:** 10.1107/S2056989015014413

**Published:** 2015-08-06

**Authors:** Rajamani Raja, Subramani Kandhasamy, Paramasivam T. Perumal, A. SubbiahPandi

**Affiliations:** a Department of Physics, Presidency College (Autonomous), Chennai 600 005, India; b Organic Chemistry Division, CSIR Central Leather Research Institute, Chennai 600 020, India

**Keywords:** crystal structure, chromene, bis­chromene, N—H⋯O hydrogen bonding, C—H⋯O hydrogen bonding

## Abstract

In the title compound, C_23_H_16_N_2_O_7_, the mean planes of the two chromene units (r.m.s. deviations = 0.031 and 0.064 Å) are almost normal to one another with a dihedral angle of 85.59 (6)°. The central six-membered pyran ring has a distorted envelope conformation, with the methine C atom at the flap. There is an intra­molecular N—H⋯O hydrogen bond, which generates an *S*(6) ring motif. In the crystal, mol­ecules are linked by pairs of N—H⋯O hydrogen bonds, forming inversion dimers with an *R*_2_^2^(12) ring motif. The dimers are linked by pairs of C—H⋯O hydrogen bonds, enclosing *R*_2_^2^(6) ring motifs, forming zigzag chains along [001]. The chains are linked by a second pair of C—H⋯O hydrogen bonds, forming slabs parallel to (110). Within the slabs there are C—H⋯π inter­actions present. A region of disordered electron density was treated with the SQUEEZE procedure in *PLATON* [Spek (2015 ▸). *Acta Cryst.* C**71**, 9–18] following unsuccessful attempts to model it as plausible solvent mol­ecule(s). The given chemical formula and other crystal data do not take into account the unknown solvent mol­ecule(s).

## Related literature   

For the uses and biological importance of chromenes, see: Ercole *et al.* (2009 ▸); Geen *et al.* (1996 ▸); Khan *et al.* (2010 ▸); Raj *et al.* (2010 ▸). For the crystal structure of a very similar compound, the 6-chloro-4-oxo-4*H*-chromen-3-yl derivative, see: Raja *et al.* (2015 ▸). 
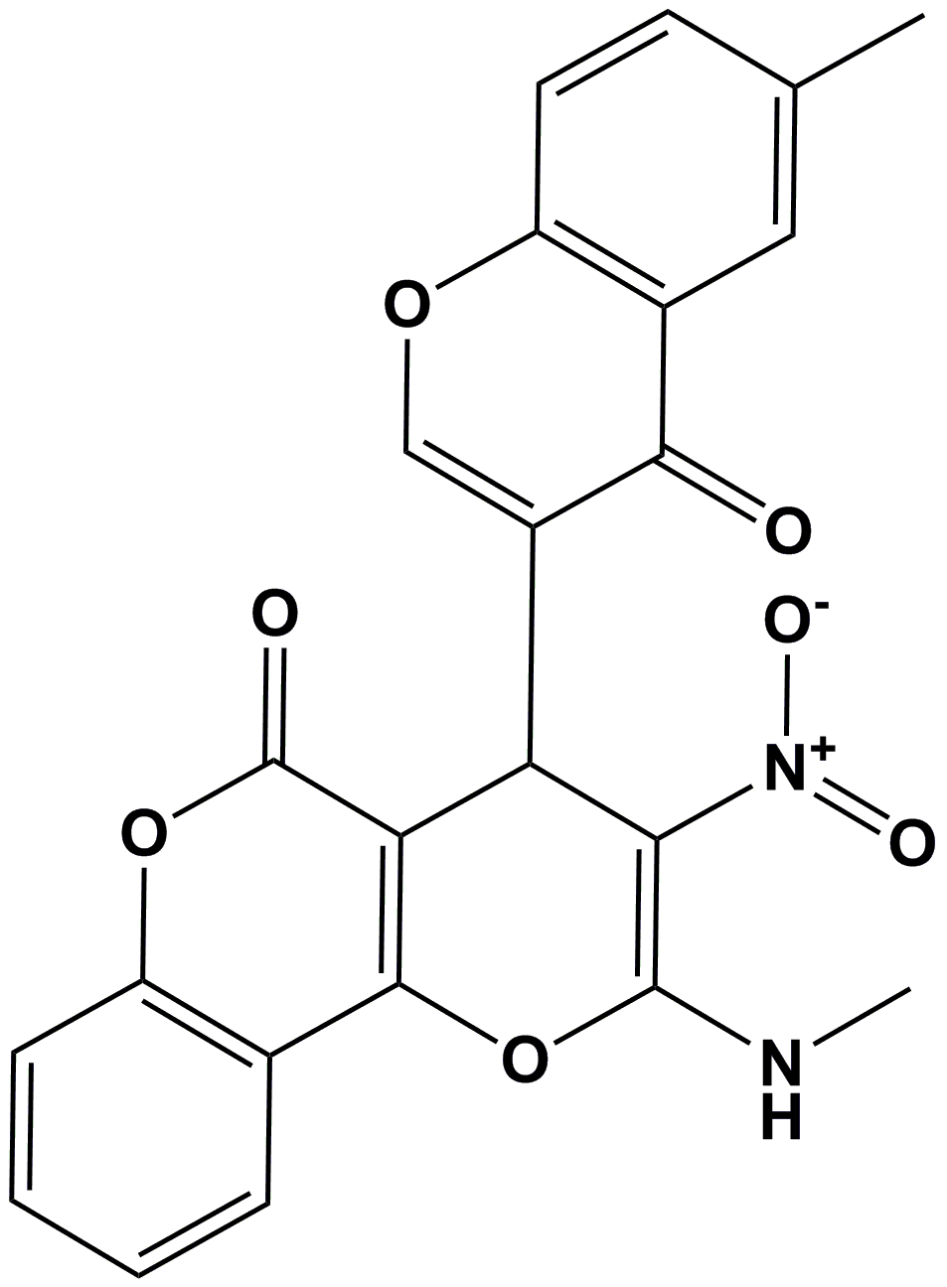


## Experimental   

### Crystal data  

C_23_H_16_N_2_O_7_
*M* *_r_* = 432.38
Triclinic, 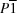

*a* = 8.0828 (2) Å
*b* = 11.2035 (3) Å
*c* = 13.4718 (3) Å
α = 68.580 (1)°
β = 78.877 (1)°
γ = 76.578 (1)°
*V* = 1096.76 (5) Å^3^
*Z* = 2
Mo *K*α radiation
μ = 0.10 mm^−1^
*T* = 293 K
0.35 × 0.30 × 0.25 mm

### Data collection  

Bruker SMART APEXII CCD diffractometer
Absorption correction: multi-scan (*SADABS*; Bruker, 2008 ▸) *T*_min_ = 0.966, *T*_max_ = 0.976
15923 measured reflections
3843 independent reflections
3371 reflections with *I* > 2σ(*I*)
*R*_int_ = 0.015

### Refinement  

*R*[*F* ^2^ > 2σ(*F* ^2^)] = 0.044
*wR*(*F* ^2^) = 0.136
*S* = 1.04
3843 reflections
295 parameters
2 restraints
H atoms treated by a mixture of independent and constrained refinement
Δρ_max_ = 0.31 e Å^−3^
Δρ_min_ = −0.25 e Å^−3^

### 

Data collection: *APEX2* (Bruker, 2008 ▸); cell refinement: *SAINT* (Bruker, 2008 ▸); data reduction: *SAINT*; program(s) used to solve structure: *SHELXS97* (Sheldrick, 2008 ▸); program(s) used to refine structure: *SHELXL97* (Sheldrick, 2008 ▸); molecular graphics: *ORTEP-3 for Windows* (Farrugia, 2012 ▸); software used to prepare material for publication: *SHELXL97* and *PLATON* (Spek, 2009 ▸).

## Supplementary Material

Crystal structure: contains datablock(s) global, I. DOI: 10.1107/S2056989015014413/su5172sup1.cif

Structure factors: contains datablock(s) I. DOI: 10.1107/S2056989015014413/su5172Isup2.hkl

Supporting information file. DOI: 10.1107/S2056989015014413/su5172Isup3.cml

**Fig. 1.**
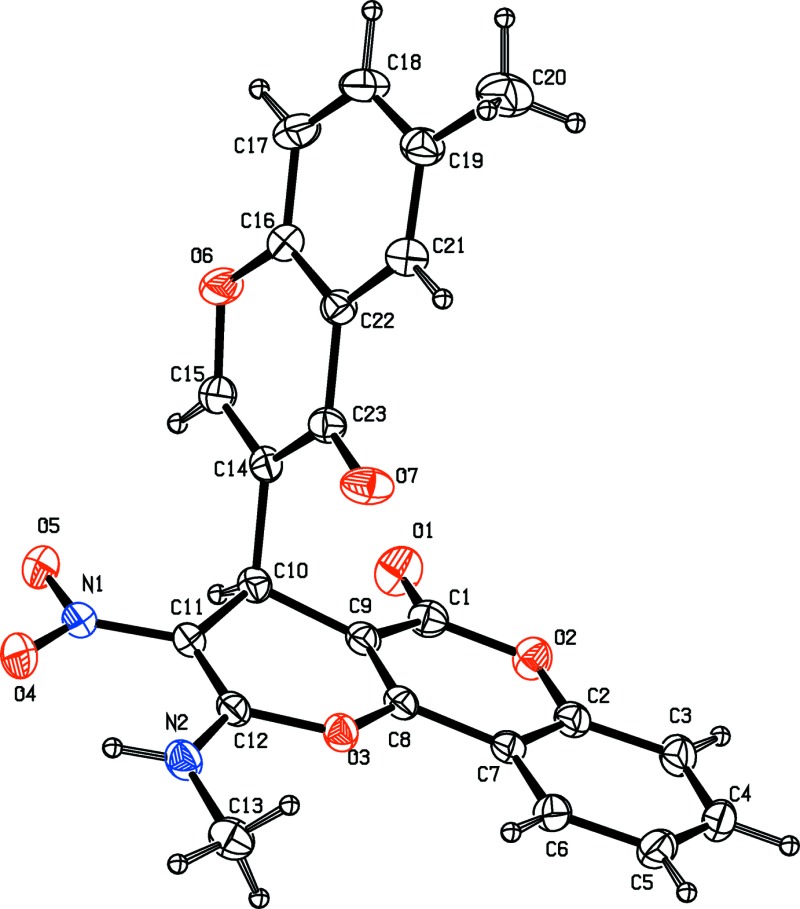
The molecular structure of the title compound, with the atom labelling. The displacement ellipsoids are drawn at 30% probability level.

**Fig. 2.**
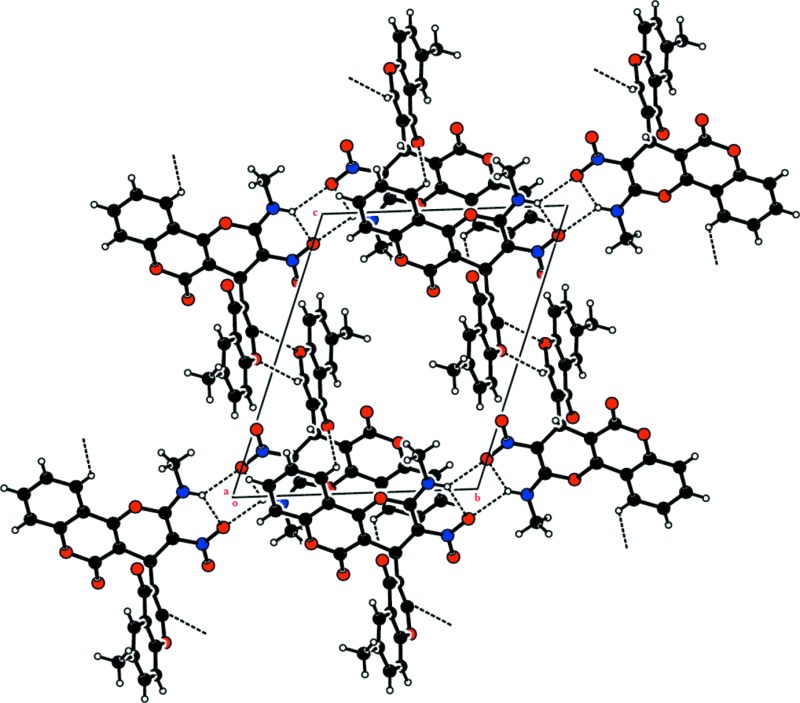
The crystal packing of the title compound, viewed along the a axis. The hydrogen bonds are shown as dashed lines (see Table 1 for details).

. DOI: 10.1107/S2056989015014413/su5172fig1.tif
The mol­ecular structure of the title compound, with the atom labelling. The displacement ellipsoids are drawn at 30% probability level.

a . DOI: 10.1107/S2056989015014413/su5172fig2.tif
The crystal packing of the title compound, viewed along the *a* axis. The hydrogen bonds are shown as dashed lines (see Table 1 for details).

CCDC reference: 1416085

Additional supporting information:  crystallographic information; 3D view; checkCIF report

## Figures and Tables

**Table 1:** Hydrogen-bond geometry (, ) *Cg*4 is the centroid of the C2C7 ring.

*D*H*A*	*D*H	H*A*	*D* *A*	*D*H*A*
N2H2*A*O4	0.89(2)	1.92(2)	2.623(2)	135(2)
N2H2*A*O4^i^	0.89(2)	2.28(2)	2.991(2)	137(2)
C15H15O6^ii^	0.93	2.55	3.255(2)	133
C6H6O7^iii^	0.93	2.51	3.136(2)	125
C13H13*B* *Cg*4^iii^	0.96	2.86	3.059(3)	142

Symmetry codes: (i) 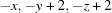; (ii) 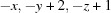; (iii) 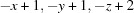.

## supplementary crystallographic information

### S1. Comment

Chromene derivatives are important heterocyclic compounds that have a variety of industrial, biological and chemical synthetic applications (Geen *et al.*, 1996; Ercole *et al.*, 2009). They exhibit a number of pharmacological activities such as anti-HIV, anti-inflammatory, anti-bacterial, anti-allergic, anti-cancer (Khan *et al.*, 2010; Raj *et al.*, 2010). Against this background, we synthesized the title compound and report herein on its crystal structure.

The molecular structure of the title compound is illustrated in Fig. 1. The mean planes of the two chromene units (O2/C1—C9; r.m.s. deviation = 0.031 Å, and O6/C14—C23; r.m.s. deviation = 0.064 Å) are almost normal to one another with a dihedral angle of 85.59 (6) °. The central six membered pyran ring (O3/C8—C12) has a distorted envelope conformation, with atom C10 at the flap. Its mean plane makes dihedral angles of 9.4 (2) and 7.3 (2) ° with the nitro (N1/O4/O5) and methyl­amine (N2/C12/C13) groups, respectively. The molecular structure is stabilized by an intra­molecular N—H···O inter­action, which generates an S(6) ring motif (Table 1). The nitro group and amine N atoms, N1 and N2, respectively, deviate by -0.270 and -0.170 Å from the mean plane of the pyran unit. Atom C20 of the methyl group deviates from the benzene ring (C16—C22) by 0.152 Å. The title compound exhibits structural similarities with a related structure, 4-(6-chloro-4-oxo-4H-chromen-3-yl)-2-methyl­amino-3-nitro-4H,5H-pyrano [3,2-c]chromen-5-one (Raja *et al.*, 2015), that crystallized as a chloro­form solvate.

In the crystal, molecules are linked by pairs of N—H···O hydrogen bonds forming inversion dimers with an *R*_2_^2^(12) ring motif. The dimers are linked by pairs of C—H···O hydrogen bonds, enclosing *R*_2_^2^(6) ring motifs, forming zigzag chains along [001]. The chains are linked by a second pair of C—H···O hydrogen bonds forming slabs parallel to (110). Within the slabs there are C—H···π inter­actions present. Details of the hydrogen bonding and other inter­actions are given in Table 1 and Fig. 2.

### S2. Synthesis and crystallization

The title compound was prepared by a three component coupling reaction in the presence of indium(III) chloride as a Lewis acid catalyst. The combination of ethanol and InCl_3_ gave an excellent result with a short reaction time. To a solution of 4-hy­droxy­coumarin (0.81 g, 5 mmol), 6-methyl-4-oxo-4*H*-chromene-3-carbaldehyde (0.97 g, 5 mmol) and NMSM (0.74 g, 5 mmol) in EtOH at room temperature was added indium(III) chloride (0.2 eq). Upon completion of the reaction (monitored by TLC) after 2 h, the mixture was filtered, and washed with ethanol to obtained the desired product (yield = 93%).

### S3. Refinement

Crystal data, data collection and structure refinement details are summarized in Table 2. The NH H atom was located in a difference Fourier map and freely refined. The C-bound H atoms were positioned geometrically and allowed to ride on their parent atoms: C–H = 0.93–0.98 Å with *U*_iso_(H) = 1.5*U*_eq_(C) for methyl H atoms and 1.2*U*_eq_(C) for other H atoms. A region of disordered electron density was treated with the SQUEEZE procedure in PLATON [Spek (2015). Acta Cryst. C71, 9-18] following unsuccessful attempts to model it as plausible solvent molecules. The given chemical formula and other crystal data do not take into account the unknown solvent molecules.

### Crystal data

**Table:**

C_23_H_16_N_2_O_7_	*Z* = 2
*M**_r_* = 432.38	*F*(000) = 448
Triclinic, *P*1	*D*_x_ = 1.309 Mg m^−^^3^
Hall symbol: -P 1	Mo *K*α radiation, λ = 0.71073 Å
*a* = 8.0828 (2) Å	Cell parameters from 3371 reflections
*b* = 11.2035 (3) Å	θ = 2.0–25.0°
*c* = 13.4718 (3) Å	µ = 0.10 mm^−^^1^
α = 68.580 (1)°	*T* = 293 K
β = 78.877 (1)°	Block, colourless
γ = 76.578 (1)°	0.35 × 0.30 × 0.25 mm
*V* = 1096.76 (5) Å^3^	

### Data collection

**Table:**

Bruker SMART APEXII CCD diffractometer	3843 independent reflections
Radiation source: fine-focus sealed tube	3371 reflections with *I* > 2σ(*I*)
Graphite monochromator	*R*_int_ = 0.015
ω and φ scans	θ_max_ = 25.0°, θ_min_ = 2.0°
Absorption correction: multi-scan (*SADABS*; Bruker, 2008)	*h* = −9→9
*T*_min_ = 0.966, *T*_max_ = 0.976	*k* = −12→13
15923 measured reflections	*l* = −16→16

### Refinement

**Table:**

Refinement on *F*^2^	Secondary atom site location: difference Fourier map
Least-squares matrix: full	Hydrogen site location: inferred from neighbouring sites
*R*[*F*^2^ > 2σ(*F*^2^)] = 0.044	H atoms treated by a mixture of independent and constrained refinement
*wR*(*F*^2^) = 0.136	*w* = 1/[σ^2^(*F*_o_^2^) + (0.072*P*)^2^ + 0.4436*P*] where *P* = (*F*_o_^2^ + 2*F*_c_^2^)/3
*S* = 1.04	(Δ/σ)_max_ < 0.001
3843 reflections	Δρ_max_ = 0.31 e Å^−^^3^
295 parameters	Δρ_min_ = −0.25 e Å^−^^3^
2 restraints	Extinction correction: *SHELXL97* (Sheldrick, 2008), Fc^*^=kFc[1+0.001xFc^2^λ^3^/sin(2θ)]^-1/4^
Primary atom site location: structure-invariant direct methods	Extinction coefficient: 0.008 (2)

### Special details

**Table:**

Geometry. All e.s.d.'s (except the e.s.d. in the dihedral angle between two l.s. planes) are estimated using the full covariance matrix. The cell e.s.d.'s are taken into account individually in the estimation of e.s.d.'s in distances, angles and torsion angles; correlations between e.s.d.'s in cell parameters are only used when they are defined by crystal symmetry. An approximate (isotropic) treatment of cell e.s.d.'s is used for estimating e.s.d.'s involving l.s. planes.
Refinement. Refinement of *F*^2^ against ALL reflections. The weighted *R*-factor *wR* and goodness of fit *S* are based on *F*^2^, conventional *R*-factors *R* are based on *F*, with *F* set to zero for negative *F*^2^. The threshold expression of *F*^2^ > σ(*F*^2^) is used only for calculating *R*-factors(gt) *etc*. and is not relevant to the choice of reflections for refinement. *R*-factors based on *F*^2^ are statistically about twice as large as those based on *F*, and *R*- factors based on ALL data will be even larger.

### Fractional atomic coordinates and isotropic or equivalent isotropic displacement parameters (Å^2^)

**Table:**

	*x*	*y*	*z*	*U*_iso_*/*U*_eq_	
C1	0.0694 (2)	0.51857 (18)	0.78511 (14)	0.0377 (4)	
C2	0.2157 (2)	0.32971 (17)	0.91324 (14)	0.0359 (4)	
C3	0.2648 (2)	0.19541 (18)	0.94755 (16)	0.0443 (5)	
H3	0.2412	0.1466	0.9107	0.053*	
C4	0.3494 (3)	0.13584 (18)	1.03749 (17)	0.0487 (5)	
H4	0.3821	0.0457	1.0618	0.058*	
C5	0.3865 (2)	0.20740 (19)	1.09226 (16)	0.0468 (5)	
H5	0.4444	0.1653	1.1525	0.056*	
C6	0.3380 (2)	0.34104 (18)	1.05799 (14)	0.0391 (4)	
H6	0.3627	0.3890	1.0952	0.047*	
C7	0.2513 (2)	0.40433 (16)	0.96706 (13)	0.0319 (4)	
C8	0.1979 (2)	0.54278 (16)	0.92247 (13)	0.0311 (4)	
C9	0.1178 (2)	0.59925 (16)	0.83416 (13)	0.0316 (4)	
C10	0.0759 (2)	0.74462 (16)	0.78257 (13)	0.0338 (4)	
H10	−0.0427	0.7676	0.7661	0.041*	
C11	0.0886 (2)	0.80769 (16)	0.86107 (13)	0.0352 (4)	
C12	0.1690 (2)	0.74174 (16)	0.95340 (13)	0.0340 (4)	
C13	0.2594 (3)	0.7133 (2)	1.12655 (16)	0.0489 (5)	
H13A	0.2585	0.7702	1.1655	0.073*	
H13B	0.3750	0.6725	1.1113	0.073*	
H13C	0.1920	0.6478	1.1690	0.073*	
C14	0.1920 (2)	0.79154 (16)	0.67790 (13)	0.0334 (4)	
C15	0.1217 (2)	0.86432 (18)	0.58849 (14)	0.0427 (4)	
H15	0.0027	0.8843	0.5940	0.051*	
C16	0.3843 (2)	0.87622 (17)	0.47969 (14)	0.0406 (4)	
C17	0.4691 (3)	0.9150 (2)	0.37663 (15)	0.0542 (5)	
H17	0.4092	0.9665	0.3187	0.065*	
C18	0.6426 (3)	0.8761 (2)	0.36163 (16)	0.0561 (6)	
H18	0.7000	0.9022	0.2925	0.067*	
C19	0.7366 (3)	0.7983 (2)	0.44682 (16)	0.0470 (5)	
C20	0.9251 (3)	0.7457 (3)	0.4270 (2)	0.0695 (7)	
H20A	0.9637	0.7790	0.3517	0.104*	
H20B	0.9430	0.6523	0.4503	0.104*	
H20C	0.9884	0.7722	0.4663	0.104*	
C21	0.6498 (2)	0.76466 (19)	0.54935 (15)	0.0416 (4)	
H21	0.7108	0.7160	0.6074	0.050*	
C22	0.4719 (2)	0.80227 (16)	0.56768 (13)	0.0348 (4)	
C23	0.3775 (2)	0.75983 (16)	0.67571 (13)	0.0347 (4)	
N1	0.0117 (2)	0.93706 (14)	0.83773 (12)	0.0421 (4)	
N2	0.1876 (2)	0.78827 (16)	1.02652 (12)	0.0416 (4)	
O1	−0.01634 (19)	0.55805 (14)	0.71221 (11)	0.0545 (4)	
O2	0.12479 (17)	0.38573 (12)	0.82583 (10)	0.0425 (3)	
O3	0.23490 (15)	0.61246 (11)	0.97862 (9)	0.0357 (3)	
O4	0.0280 (2)	1.00121 (13)	0.89445 (11)	0.0578 (4)	
O5	−0.07417 (19)	0.98938 (13)	0.76059 (11)	0.0537 (4)	
O6	0.20862 (17)	0.91158 (14)	0.49046 (10)	0.0510 (4)	
O7	0.44855 (17)	0.70098 (15)	0.75704 (10)	0.0510 (4)	
H2A	0.138 (3)	0.8717 (17)	1.012 (2)	0.069 (7)*	

### Atomic displacement parameters (Å^2^)

**Table:**

	*U*^11^	*U*^22^	*U*^33^	*U*^12^	*U*^13^	*U*^23^
C1	0.0364 (9)	0.0412 (10)	0.0370 (9)	−0.0099 (7)	−0.0025 (7)	−0.0139 (8)
C2	0.0314 (9)	0.0366 (9)	0.0385 (9)	−0.0073 (7)	0.0029 (7)	−0.0140 (7)
C3	0.0453 (11)	0.0355 (9)	0.0533 (11)	−0.0100 (8)	0.0039 (9)	−0.0195 (8)
C4	0.0480 (11)	0.0290 (9)	0.0575 (12)	−0.0033 (8)	0.0044 (9)	−0.0086 (8)
C5	0.0436 (11)	0.0407 (10)	0.0436 (10)	−0.0028 (8)	−0.0042 (8)	−0.0033 (8)
C6	0.0373 (9)	0.0385 (9)	0.0375 (9)	−0.0052 (7)	−0.0026 (7)	−0.0099 (7)
C7	0.0273 (8)	0.0333 (8)	0.0329 (8)	−0.0060 (7)	0.0032 (6)	−0.0117 (7)
C8	0.0274 (8)	0.0345 (9)	0.0328 (8)	−0.0061 (7)	0.0019 (6)	−0.0153 (7)
C9	0.0277 (8)	0.0348 (9)	0.0319 (8)	−0.0053 (7)	0.0013 (6)	−0.0131 (7)
C10	0.0310 (8)	0.0343 (9)	0.0333 (9)	−0.0005 (7)	−0.0030 (7)	−0.0117 (7)
C11	0.0366 (9)	0.0311 (9)	0.0346 (9)	−0.0029 (7)	0.0043 (7)	−0.0133 (7)
C12	0.0313 (8)	0.0337 (9)	0.0377 (9)	−0.0074 (7)	0.0064 (7)	−0.0171 (7)
C13	0.0514 (11)	0.0580 (12)	0.0448 (11)	−0.0123 (9)	−0.0050 (9)	−0.0249 (9)
C14	0.0363 (9)	0.0304 (8)	0.0331 (9)	−0.0015 (7)	−0.0036 (7)	−0.0131 (7)
C15	0.0387 (10)	0.0408 (10)	0.0378 (9)	0.0030 (8)	−0.0032 (8)	−0.0074 (8)
C16	0.0452 (10)	0.0344 (9)	0.0367 (9)	−0.0037 (8)	−0.0025 (8)	−0.0086 (7)
C17	0.0632 (13)	0.0530 (12)	0.0317 (10)	−0.0079 (10)	−0.0030 (9)	−0.0001 (9)
C18	0.0653 (14)	0.0600 (13)	0.0352 (10)	−0.0206 (11)	0.0125 (9)	−0.0105 (9)
C19	0.0474 (11)	0.0495 (11)	0.0456 (11)	−0.0181 (9)	0.0077 (8)	−0.0183 (9)
C20	0.0481 (13)	0.0887 (18)	0.0642 (14)	−0.0198 (12)	0.0122 (11)	−0.0219 (13)
C21	0.0404 (10)	0.0474 (10)	0.0377 (9)	−0.0113 (8)	−0.0031 (8)	−0.0139 (8)
C22	0.0404 (9)	0.0332 (9)	0.0316 (9)	−0.0082 (7)	−0.0026 (7)	−0.0117 (7)
C23	0.0378 (9)	0.0378 (9)	0.0302 (8)	−0.0066 (7)	−0.0054 (7)	−0.0129 (7)
N1	0.0500 (9)	0.0327 (8)	0.0379 (8)	−0.0046 (7)	0.0067 (7)	−0.0128 (7)
N2	0.0468 (9)	0.0403 (9)	0.0428 (9)	−0.0071 (7)	−0.0010 (7)	−0.0225 (7)
O1	0.0614 (9)	0.0560 (9)	0.0524 (8)	−0.0106 (7)	−0.0247 (7)	−0.0163 (7)
O2	0.0503 (8)	0.0375 (7)	0.0454 (7)	−0.0108 (6)	−0.0092 (6)	−0.0168 (6)
O3	0.0401 (7)	0.0339 (6)	0.0353 (6)	−0.0029 (5)	−0.0064 (5)	−0.0156 (5)
O4	0.0834 (11)	0.0369 (7)	0.0536 (8)	−0.0042 (7)	0.0007 (7)	−0.0240 (7)
O5	0.0635 (9)	0.0379 (7)	0.0459 (8)	0.0067 (6)	−0.0068 (7)	−0.0075 (6)
O6	0.0469 (8)	0.0526 (8)	0.0336 (7)	0.0055 (6)	−0.0062 (6)	0.0006 (6)
O7	0.0394 (7)	0.0776 (10)	0.0307 (7)	−0.0093 (7)	−0.0087 (5)	−0.0104 (6)

### Geometric parameters (Å, º)

**Table:**

C1—O1	1.201 (2)	C13—H13A	0.9600
C1—O2	1.380 (2)	C13—H13B	0.9600
C1—C9	1.452 (2)	C13—H13C	0.9600
C2—O2	1.379 (2)	C14—C15	1.331 (2)
C2—C3	1.386 (3)	C14—C23	1.455 (2)
C2—C7	1.394 (2)	C15—O6	1.350 (2)
C3—C4	1.378 (3)	C15—H15	0.9300
C3—H3	0.9300	C16—O6	1.377 (2)
C4—C5	1.381 (3)	C16—C17	1.385 (3)
C4—H4	0.9300	C16—C22	1.388 (2)
C5—C6	1.379 (3)	C17—C18	1.367 (3)
C5—H5	0.9300	C17—H17	0.9300
C6—C7	1.400 (2)	C18—C19	1.397 (3)
C6—H6	0.9300	C18—H18	0.9300
C7—C8	1.436 (2)	C19—C21	1.382 (3)
C8—C9	1.339 (2)	C19—C20	1.510 (3)
C8—O3	1.378 (2)	C20—H20A	0.9600
C9—C10	1.503 (2)	C20—H20B	0.9600
C10—C11	1.500 (2)	C20—H20C	0.9600
C10—C14	1.524 (2)	C21—C22	1.401 (3)
C10—H10	0.9800	C21—H21	0.9300
C11—N1	1.382 (2)	C22—C23	1.470 (2)
C11—C12	1.390 (2)	C23—O7	1.227 (2)
C12—N2	1.316 (2)	N1—O5	1.245 (2)
C12—O3	1.360 (2)	N1—O4	1.266 (2)
C13—N2	1.451 (3)	N2—H2A	0.891 (17)
			
O1—C1—O2	117.20 (16)	H13B—C13—H13C	109.5
O1—C1—C9	125.20 (17)	C15—C14—C23	120.17 (16)
O2—C1—C9	117.59 (15)	C15—C14—C10	119.15 (15)
O2—C2—C3	117.04 (16)	C23—C14—C10	120.67 (14)
O2—C2—C7	121.46 (15)	C14—C15—O6	125.45 (17)
C3—C2—C7	121.46 (17)	C14—C15—H15	117.3
C4—C3—C2	118.48 (18)	O6—C15—H15	117.3
C4—C3—H3	120.8	O6—C16—C17	116.87 (17)
C2—C3—H3	120.8	O6—C16—C22	121.59 (15)
C3—C4—C5	121.26 (17)	C17—C16—C22	121.51 (18)
C3—C4—H4	119.4	C18—C17—C16	118.79 (19)
C5—C4—H4	119.4	C18—C17—H17	120.6
C6—C5—C4	120.26 (18)	C16—C17—H17	120.6
C6—C5—H5	119.9	C17—C18—C19	122.10 (18)
C4—C5—H5	119.9	C17—C18—H18	119.0
C5—C6—C7	119.81 (18)	C19—C18—H18	119.0
C5—C6—H6	120.1	C21—C19—C18	117.97 (19)
C7—C6—H6	120.1	C21—C19—C20	120.9 (2)
C2—C7—C6	118.73 (15)	C18—C19—C20	121.04 (18)
C2—C7—C8	116.42 (15)	C19—C20—H20A	109.5
C6—C7—C8	124.85 (16)	C19—C20—H20B	109.5
C9—C8—O3	122.85 (15)	H20A—C20—H20B	109.5
C9—C8—C7	122.76 (15)	C19—C20—H20C	109.5
O3—C8—C7	114.38 (14)	H20A—C20—H20C	109.5
C8—C9—C1	119.56 (15)	H20B—C20—H20C	109.5
C8—C9—C10	122.40 (15)	C19—C21—C22	121.44 (18)
C1—C9—C10	118.05 (14)	C19—C21—H21	119.3
C11—C10—C9	108.94 (14)	C22—C21—H21	119.3
C11—C10—C14	111.95 (14)	C16—C22—C21	118.12 (16)
C9—C10—C14	111.16 (13)	C16—C22—C23	120.13 (16)
C11—C10—H10	108.2	C21—C22—C23	121.67 (16)
C9—C10—H10	108.2	O7—C23—C14	122.72 (15)
C14—C10—H10	108.2	O7—C23—C22	123.01 (16)
N1—C11—C12	120.88 (15)	C14—C23—C22	114.27 (14)
N1—C11—C10	115.91 (15)	O5—N1—O4	120.53 (15)
C12—C11—C10	123.20 (14)	O5—N1—C11	119.20 (15)
N2—C12—O3	112.04 (15)	O4—N1—C11	120.28 (16)
N2—C12—C11	127.77 (16)	C12—N2—C13	125.94 (16)
O3—C12—C11	120.16 (14)	C12—N2—H2A	112.1 (16)
N2—C13—H13A	109.5	C13—N2—H2A	121.6 (16)
N2—C13—H13B	109.5	C2—O2—C1	121.84 (14)
H13A—C13—H13B	109.5	C12—O3—C8	119.57 (13)
N2—C13—H13C	109.5	C15—O6—C16	117.95 (14)
H13A—C13—H13C	109.5		
			
O2—C2—C3—C4	−177.26 (16)	C10—C14—C15—O6	−179.49 (17)
C7—C2—C3—C4	0.5 (3)	O6—C16—C17—C18	−176.57 (19)
C2—C3—C4—C5	−0.6 (3)	C22—C16—C17—C18	1.8 (3)
C3—C4—C5—C6	0.4 (3)	C16—C17—C18—C19	0.2 (3)
C4—C5—C6—C7	−0.2 (3)	C17—C18—C19—C21	−2.4 (3)
O2—C2—C7—C6	177.36 (14)	C17—C18—C19—C20	173.9 (2)
C3—C2—C7—C6	−0.3 (2)	C18—C19—C21—C22	2.7 (3)
O2—C2—C7—C8	−3.8 (2)	C20—C19—C21—C22	−173.63 (19)
C3—C2—C7—C8	178.49 (15)	O6—C16—C22—C21	176.78 (16)
C5—C6—C7—C2	0.2 (2)	C17—C16—C22—C21	−1.5 (3)
C5—C6—C7—C8	−178.54 (16)	O6—C16—C22—C23	−0.1 (3)
C2—C7—C8—C9	0.1 (2)	C17—C16—C22—C23	−178.40 (18)
C6—C7—C8—C9	178.86 (15)	C19—C21—C22—C16	−0.8 (3)
C2—C7—C8—O3	179.64 (13)	C19—C21—C22—C23	176.05 (17)
C6—C7—C8—O3	−1.6 (2)	C15—C14—C23—O7	174.75 (17)
O3—C8—C9—C1	−174.38 (14)	C10—C14—C23—O7	−3.9 (3)
C7—C8—C9—C1	5.1 (2)	C15—C14—C23—C22	−6.3 (2)
O3—C8—C9—C10	5.9 (2)	C10—C14—C23—C22	175.04 (14)
C7—C8—C9—C10	−174.58 (14)	C16—C22—C23—O7	−175.61 (17)
O1—C1—C9—C8	172.77 (17)	C21—C22—C23—O7	7.6 (3)
O2—C1—C9—C8	−6.7 (2)	C16—C22—C23—C14	5.4 (2)
O1—C1—C9—C10	−7.5 (3)	C21—C22—C23—C14	−171.34 (16)
O2—C1—C9—C10	173.01 (14)	C12—C11—N1—O5	−172.65 (16)
C8—C9—C10—C11	−16.7 (2)	C10—C11—N1—O5	6.1 (2)
C1—C9—C10—C11	163.63 (14)	C12—C11—N1—O4	7.1 (3)
C8—C9—C10—C14	107.12 (17)	C10—C11—N1—O4	−174.13 (15)
C1—C9—C10—C14	−72.56 (19)	O3—C12—N2—C13	−3.6 (2)
C9—C10—C11—N1	−163.56 (14)	C11—C12—N2—C13	174.31 (17)
C14—C10—C11—N1	73.10 (19)	C3—C2—O2—C1	179.89 (15)
C9—C10—C11—C12	15.1 (2)	C7—C2—O2—C1	2.1 (2)
C14—C10—C11—C12	−108.19 (18)	O1—C1—O2—C2	−176.34 (16)
N1—C11—C12—N2	−1.8 (3)	C9—C1—O2—C2	3.1 (2)
C10—C11—C12—N2	179.60 (17)	N2—C12—O3—C8	167.61 (14)
N1—C11—C12—O3	175.96 (14)	C11—C12—O3—C8	−10.4 (2)
C10—C11—C12—O3	−2.7 (2)	C9—C8—O3—C12	9.0 (2)
C11—C10—C14—C15	−111.88 (18)	C7—C8—O3—C12	−170.52 (13)
C9—C10—C14—C15	126.04 (17)	C14—C15—O6—C16	4.0 (3)
C11—C10—C14—C23	66.81 (19)	C17—C16—O6—C15	173.66 (18)
C9—C10—C14—C23	−55.3 (2)	C22—C16—O6—C15	−4.7 (3)
C23—C14—C15—O6	1.8 (3)		

### Hydrogen-bond geometry (Å, º)

Cg4 is the centroid of the C2–C7 ring.

**Table:**

*D*—H···*A*	*D*—H	H···*A*	*D*···*A*	*D*—H···*A*
N2—H2*A*···O4	0.89 (2)	1.92 (2)	2.623 (2)	135 (2)
N2—H2*A*···O4^i^	0.89 (2)	2.28 (2)	2.991 (2)	137 (2)
C15—H15···O6^ii^	0.93	2.55	3.255 (2)	133
C6—H6···O7^iii^	0.93	2.51	3.136 (2)	125
C13—H13*B*···*Cg*4^iii^	0.96	2.86	3.059 (3)	142

Symmetry codes: (i) −*x*, −*y*+2, −*z*+2; (ii) −*x*, −*y*+2, −*z*+1; (iii) −*x*+1, −*y*+1, −*z*+2.
