# Supplementary figures and images for: Crystal structure of 2-methyl­amino-4-(6-methyl-4-oxo-4H-chromen-3-yl)-3-nitro­pyrano[3,2-c]chromen-5(4H)-one with an unknown solvate
Source: Acta Crystallogr E Crystallogr Commun. 2015 Aug 6;71(Pt 9):o645–6. doi: 10.1107/S2056989015014413 (PMC4555399; doi:10.1107/S2056989015014413)

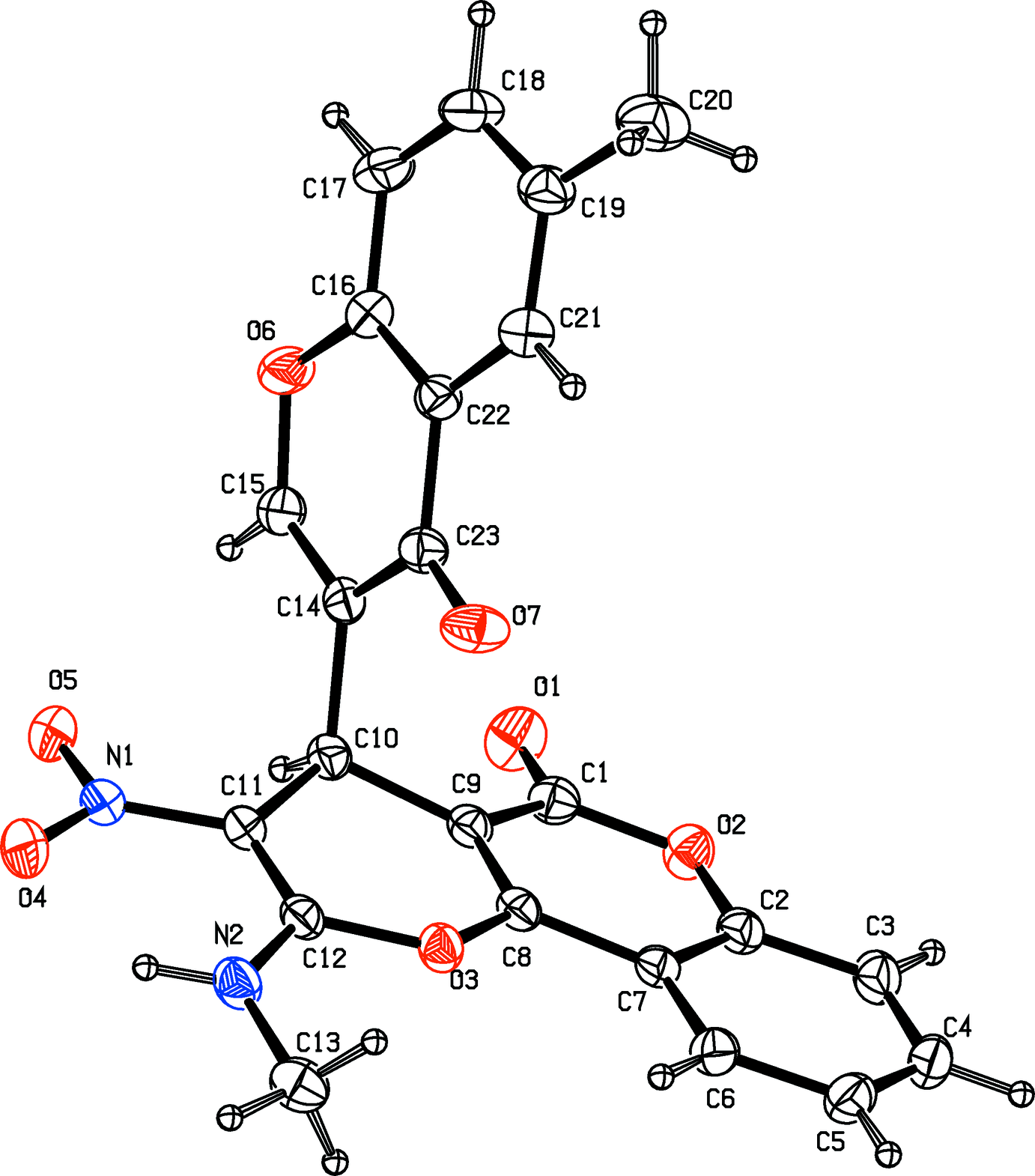

Supplement: Supplementary file 4 [file e-71-0o645-fig1.tif]

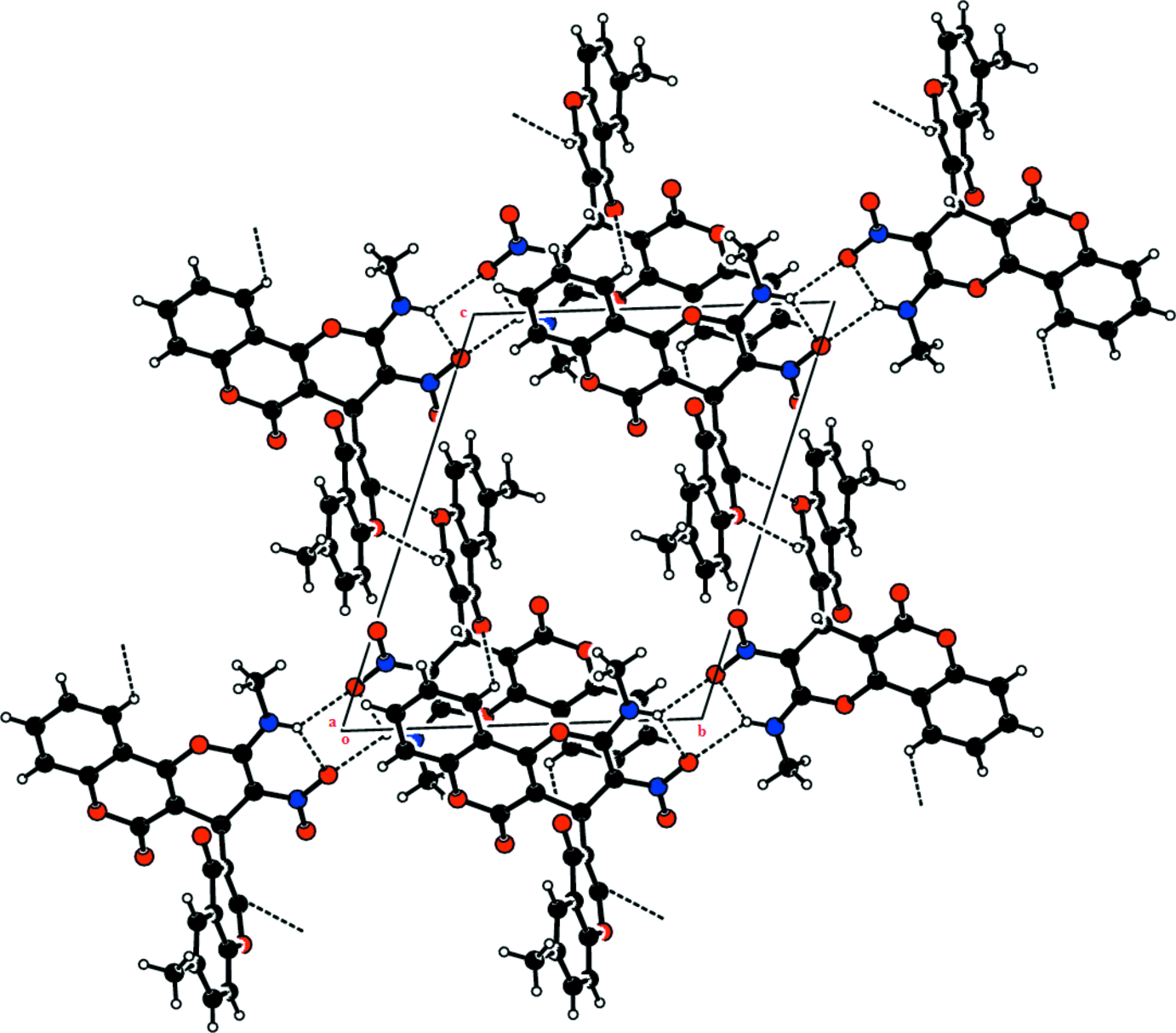

Supplement: Supplementary file 5 [file e-71-0o645-fig2.tif]
